# Plasma Cell-Free DNA Methylomics of Bipolar Disorder With and Without Rapid Cycling

**DOI:** 10.3389/fnins.2021.774037

**Published:** 2021-11-30

**Authors:** Ada Man-Choi Ho, Stacey J. Winham, Bryan M. McCauley, Marija Kundakovic, Keith D. Robertson, Zhifu Sun, Tamas Ordog, Lauren M. Webb, Mark A. Frye, Marin Veldic

**Affiliations:** ^1^Department of Psychiatry and Psychology, Mayo Clinic, Rochester, MN, United States; ^2^Department of Health Science Research, Mayo Clinic, Rochester, MN, United States; ^3^Department of Biological Sciences, Fordham University, New York, NY, United States; ^4^Department of Molecular Pharmacology and Experimental Therapeutics, Mayo Clinic, Rochester, MN, United States; ^5^Department of Physiology and Biomedical Engineering, Mayo Clinic, Rochester, MN, United States; ^6^Mayo Clinic Alix School of Medicine, Rochester, MN, United States

**Keywords:** bipolar disorder, rapid cycling, plasma, cell-free DNA, methylomics, microarray

## Abstract

Rapid cycling (RC) burdens bipolar disorder (BD) patients further by causing more severe disability and increased suicidality. Because diagnosing RC can be challenging, RC patients are at risk of rapid decline due to delayed suitable treatment. Here, we aimed to identify the differences in the circulating cell-free DNA (cfDNA) methylome between BD patients with and without RC. The cfDNA methylome could potentially be developed as a diagnostic test for BD RC. We extracted cfDNA from plasma samples of BD1 patients (46 RC and 47 non-RC). cfDNA methylation levels were measured by 850K Infinium MethylationEPIC array. Principal component analysis (PCA) was conducted to assess global differences in methylome. cfDNA methylation levels were compared between RC groups using a linear model adjusted for age and sex. PCA suggested differences in methylation profiles between RC groups (*p* = 0.039) although no significant differentially methylated probes (DMPs; *q* > 0.15) were found. The top four CpG sites which differed between groups at *p* < 1E-05 were located in *CGGPB1*, *PEX10*, *NR0B2*, and *TP53I11*. Gene set enrichment analysis (GSEA) on top DMPs (*p* < 0.05) showed significant enrichment of gene sets related to nervous system tissues, such as neurons, synapse, and glutamate neurotransmission. Other top notable gene sets were related to parathyroid regulation and calcium signaling. To conclude, our study demonstrated the feasibility of utilizing a microarray method to identify circulating cfDNA methylation sites associated with BD RC and found the top differentially methylated CpG sites were mostly related to the nervous system and the parathyroid.

## Introduction

Bipolar disorder (BD) features recurrent episodes of mania/hypomania and depression, interspersed with periods of euthymia. Symptoms usually include drastic changes in energy levels, sleep, thinking, and behaviors, which can significantly disrupt the daily life of BD patients (Craddock and Sklar, 2013). A mood cycle is defined as the period from the onset of a mood episode of any polarity to the emergence of another mood episode of any polarity. Mood cycles could last for weeks to months. When four or more distinct mood episodes (manic, hypomanic, major depressive, or mixed) occur within 12 months, demarcated by a period of partial or full remission for a least 2 months or by a switch to an episode of the opposite polarity, the phenomenon is specified as *rapid cycling* (American Psychiatric Association, 2000). Rapid cycling affects a significant proportion of BD patients with a lifetime prevalence of 33%, particularly in those with a longer course of illness, early onset age, and substance abuse (Fountoulakis et al., 2013; Gigante et al., 2016). It has been suggested that rapid cycling represents a decline of the disorder; it is associated with more severe disability and increased suicidality and demands different treatment strategies (Fountoulakis et al., 2013). However, the diagnosis of rapid cycling may be difficult since not all rapid cyclers achieve full remissions between episodes, which may lead to misdiagnosis such as borderline personality disorder (Mackinnon and Pies, 2006) and delay appropriate treatment.

The etiology of rapid mood cycling is hitherto elusive. Female sex, BD type II, and antidepressant treatment have been associated with the risk of rapid cycling (Fountoulakis et al., 2013). Increased susceptibility to DNA damage, hypothyroidism, and insulin resistance had also been observed in BD patients with the rapid cycling feature. However, it remains unclear whether these factors are the causes of rapid cycling or the consequences of drug treatment or medical comorbidities (Buoli et al., 2017). Our group recently reported a genetic association between the glutamate transporter 2 gene *SLC1A2* and rapid cycling in a group of ∼2,000 depressed patients (Veldic et al., 2019). We found the minor allele of the single nucleotide polymorphism (SNP) rs3812778, located downstream of *SLC1A2*, was associated with an increased risk of rapid cycling as well as increased anterior cingulate glutamate levels, implicating genetic effects on the regulation of mood fluctuation via the synaptic clearance of glutamate in this brain region (Veldic et al., 2019). While genetics informs the risks of BD and related features, state-dependent biomarkers that are associated with mood instability may help the prognosis and diagnosis of rapid cycling thus need to be identified.

Circulating cell-free DNA (cfDNA) present in plasma, urine, cerebrospinal fluid, and other bodily fluids are short DNA fragments believed to be derived from cells undergoing apoptosis and necrosis and, possibly, released by active secretion (Wan et al., 2017). In healthy individuals, cfDNA concentration is generally low and the majority of plasma cfDNA is released from cells of hematopoietic lineage and to a lesser extent from other tissues (Lui et al., 2002; Sun et al., 2015; Moss et al., 2018), but in certain physiological and clinical conditions, such as exercise, pregnancy, infection, acute trauma, and transplantation, cfDNA concentration and/or composition (in terms of the tissue/cell/donor of origin) were found to be altered (Grace et al., 2016; Wan et al., 2017; Goh et al., 2018), thus allowing cfDNA to become a potential biomarker of various conditions and diseases. For example, screening of cfDNA in maternal serum for aneuploidy and other genetic conditions of the fetus (by detecting placenta-derived cfDNA) is now widely applied (Grace et al., 2016). Serum or plasma cfDNA has demonstrated prognostic and diagnostic potential in various cancers by detecting tumor-derived cfDNA (Wan et al., 2017). Since alterations in DNA methylation have been observed in brain tissues and related cell types of various psychiatric illnesses, including posttraumatic stress disorder (Blacker et al., 2019), schizophrenia (Veldic et al., 2004; Costa et al., 2006; Guidotti et al., 2016), and BD (Guidotti et al., 2016; Ho et al., 2019), it has been proposed that DNA methylation may be involved in the etiology and progression of these illnesses. Methylation patterns in plasma cfDNA may help to determine the cell or tissue of origin (Moss et al., 2018; Huang and Wang, 2019; Liu et al., 2019). In psychiatry, recent reports on altered cfDNA abundance and methylation level after psychosocial stressor challenge (Hummel et al., 2018) and increased plasma cell-free mitochondrial DNA (cfmtDNA) in suicide attempters (Lindqvist et al., 2016) suggest cfDNA and cfmtDNA could serve as biomarkers for psychological stress response, suicidal behavior and/or depression. Since rapid cycling could be difficult to diagnose and demands immediate personalized treatment strategies to prevent further decline and to minimize the risk of suicide, the use of biomarkers would facilitate timely prescription of treatment for BD patients with rapid cycling. We conducted this study to explore the differences in the methylomic landscape of plasma cfDNA between BD patients with and without rapid cycling as foundational work for identifying BD rapid cycling diagnostic biomarkers with the use of a microarray method for cfDNA methylation detection. We hypothesized that BD patients with rapid cycling would have a different cfDNA methylomic pattern compared to BD patients without rapid cycling, which might reflect certain physiological vulnerabilities associated with this specific BD feature.

## Materials and Methods

### Subjects

This study included 93 participants enrolled in the Mayo Clinic Bipolar Disorder Biobank (Frye et al., 2015). The biobank was approved by the Mayo Clinic Institutional Review Board, and recruited patients aged 18–80 years old with a Diagnostic and Statistical Manual of Mental Disorders 4th Edition Text Revision (DSM-IV-TR) or DSM-5 diagnosis of bipolar I, bipolar II, bipolar NOS, or schizoaffective bipolar type. All subjects provided written informed consent for use of their data and biospecimens in future studies. Exclusion criteria included inability or unwillingness to provide written informed consent, active suicidal ideation, or active psychosis. Clinical phenotypes were confirmed by the Structured Clinical Interview for DSM-IV (First et al., 1997). Subjects included in this study had a diagnosis of BD1 in which 46 subjects had experienced rapid mood cycling (≥4 distinct mood episodes within a 12-month period) while 47 subjects who did not have such experience were regarded as non-rapid cycling controls.

### Plasma Cell-Free DNA Extraction

Venous blood sample was collected in potassium EDTA tubes and was centrifuged at 145 *g* for 12 min with soft deceleration. The bottom 1/3 of plasma was further centrifuged at 2,300 *g* for 10 min, only the top 3/4 of resultant plasma was collected as platelet-poor plasma which was stored at −80°C until cfDNA extraction by QIAamp Circulating Nucleic Acid Kit (Qiagen, Hilden, Germany) according to the manufacturer’s instruction.

### Genome-Wide Methylome Profiling of Plasma cfDNA by 850K MethylEPIC Array

Methylation analysis methods applied were the same as in Ho et al. (2019). About 25 ng of cfDNA was used for bisulfite conversion and methylome microarray analysis performed at the University of Minnesota Genomics Center in a single batch. Genome-wide methylomic profiling was performed on the 850K Infinium MethylationEPIC BeadChip platform (Illumina Inc., San Diego, CA, United States). Unmethylated and methylated CpGenome controls and replicates of a reference cfDNA sample were included in each array to assess inter-array consistency.

### Data Processing

Data quality control and statistical analyses were performed in R software v.3.4.1 with Bioconductor package *minfi* (Aryee et al., 2014). No sample was excluded due to low call rates (detection *p* < 0.05, apart from one rapid cycling subject’s sample with *p* = 0.07). Considering subject samples only, one sample had a locus detection rate > 99%, 66 samples had between 95 and 98.99%, 22 samples had between 90 and 94.99%, and four samples had < 89.99% (lowest 88.41%). Probes that failed in one or more samples, had a single nucleotide polymorphism at the CpG site, or mapped to non-specific genomic locations were removed. The probes located on X and Y chromosomes were excluded from data quality control procedures but included in subsequent statistical analyses. The initial methylation data contained 865 859 probes. After quality control, 463 050 probes were available for analysis (see Supplementary Method for the numbers of probes dropped for specific reasons). Data were normalized by functional normalization (*preprocessFunnorm* command in *minfi*) which removes between-array variation by regressing out variability in the control probes on each array (Fortin et al., 2014). Probes on X and Y chromosomes were normalized according to the sex of the sample.

### Multivariate Analyses

Principal component analysis (PCA) was performed on the top 2,000 most variable CpG probes considering all samples (CpG probes with the largest standard deviations in *M*-values) but probes located on X and Y chromosomes were excluded. Association between the clusters and clinical factors were tested by chi-square and Fisher’s exact tests for categorical variables and *t*-tests for continuous variables.

### Estimation of Cell-Type Composition in cfDNA Samples

We used the cell-type methylomic deconvolution algorithm and reference atlas by Moss et al. (2018) to estimate the cell-type origin composition in each subject’s plasma cfDNA sample. Briefly, the method uses non-negative least squares linear regression to determine the relative contributions of various cell types to the plasma cfDNA sample. The cell-type methylomic reference atlas includes ∼8,000 CpGs which are uniquely hypermethylated and hypomethylated in a certain tissue or cell type relative to the others (a total of 26 human cell types and tissues) (Moss et al., 2018).

### Identification of Differentially Methylated Positions

Differentially methylated positions (DMPs; methylation status at individual sites) were identified by the R Bioconductor package *limma* (Ritchie et al., 2015). Differential methylation analyses were performed comparing rapid cyclers and non-rapid cyclers using standard *limma* workflow with unpaired contrasts with age and sex as covariates. *M*-values were used for statistical analysis and β-values were reported for interpretability. Statistically significant DMP was considered at FDR-adjusted *p* (*q*-value) < 0.15 based on our previous publication using the same microarray platform (Ho et al., 2019).

### Identification of Differentially Methylated Regions

Differentially methylated regions (DMRs; methylation status across a genomic region) were identified by the R Bioconductor package *DMRcate* (Peters et al., 2015). A DMR was annotated when a region having at least two CpG sites with FDR-adjusted *p*-values at default detection level within a lambda of 220 (since the lengths of most cfDNA fragments range between 120 and 220 bp). Statistically significant DMR between groups was considered at Stouffer’s *p* < 0.05 (Ho et al., 2019).

### Gene Set Enrichment Analysis

Gene set enrichment analysis (GSEA) to determine if methylation levels of any gene sets were significantly associated with rapid cycling was performed using the *missMethyl* Bioconductor R package (Phipson et al., 2016). The analysis was performed on the DMPs at *p* < 0.05 using the Gene Ontology (GO) and Kyoto Encyclopedia of Genes and Genomes (KEGG) gene sets (Ashburner et al., 2000; Kanehisa et al., 2012). Statistically significant enrichment was considered at FDR < 0.05.

## Results

### Subject Characteristics

Subject characteristics are presented in Table 1. Rapid cyclers and non-rapid cyclers were not significantly different in terms of age, sex, onset age, substance abuse and dependence history, the use of lithium, mood stabilizers, and antidepressants, and the proportion of diabetic subjects. A significantly higher percentage of subjects were on antipsychotics in the rapid cycling group than the non-rapid cycling group (65.2% vs. 40.4%).

**TABLE 1:** Subject characteristics.

	Non-rapid cyclers (*n* = 47)	Rapid cyclers (*n* = 46)	*p-*value
Age (years ± SD)	46.3 ± 16.7	44.4 ± 16.5	0.582
Male (*n*;%)	20 (42.6%)	18 (39.1%)	0.737
Bipolar Type I (*n*;%)	47 (100%)	46 (100%)	–
**Bipolar onset age**			
≤19	10*	11	0.347
20–49	29	33	
50–64	5	1	
65–79	2	1	
**Substance dependence Hx (*n*;%)^†^**			
Alcohol	14 (31.1%)	14 (31.8%)	0.943
Nicotine	17 (37.8%)	18 (39.1%)	0.895
**Substance abuse Hx (*n*;%)^†^**			
Cocaine^†^	5 (11.1%)	4 (9.1%)	0.752
Methamphetamine^‡^	3 (6.7%)	3 (6.7%)	1.000
Heroin	0	0	–
Narcotics^‡^	3 (6.7%)	5 (10.9%)	0.479
Marijuana^‡^	8 (17.8%)	4 (8.9%)	0.215
**Psychotropic medications (*n*;%)**			
Lithium	17 (36.2%)	15 (32.6%)	0.718
Mood stabilizers	19 (40.4%)	24 (52.5%)	0.256
Antipsychotics	19 (40.4%)	30 (65.2%)	0.017
Antidepressants	22 (46.8%)	25 (54.3%)	0.467
Diabetes (*n*;%)	8 (17.0%)	4 (8.7%)	0.355

*^†^Based on data on 45 non-rapid cyclers and 44 rapid cyclers.*

*^‡^Based on data on 45 non-rapid cyclers and 45 rapid cyclers.*

*One subject with unknown bipolar onset age.

### Multivariate Analysis

**FIGURE 1:**
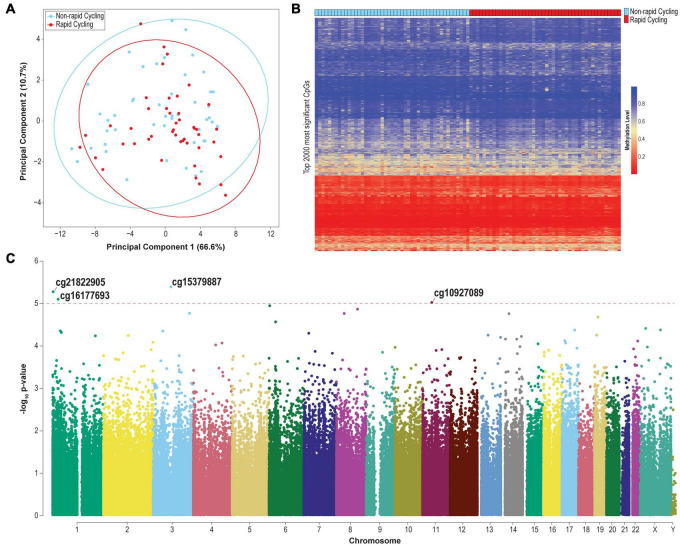
**(A)** PCA plot based on the top 2,000 most variable probes (CpG sites located on chromosomes X and Y were excluded). **(B)** Heatmap of the methylation levels of the top 2,000 most significant differentially methylated probes. **(C)** Manhattan plot of genome-wide plasma cfDNA methylomic comparison between rapid cyclers and non-rapid cyclers among bipolar disorder patients. The CpG probes where the top differentially methylated sites (*p* < 1E-05) are located are indicated. Red line: *p* = 1E-05.

PCA identified a remarkable percentage of variance explained by PC1, which overshadowed the variance explained by other PCs (Figure 1A). The clustering of samples according to PC1 and PC2 was not significantly associated with technical factors (sample plate and array), sex, age, and substance abuse/dependence history (*p* > 0.05). No significant clustering was observed by substance abuse/dependence history (Supplementary Figure 1) or psychotropic medication class (Supplementary Figure 2).

The clustering of samples according to PC2 was significantly associated with rapid cycling (Kruskal-Wallis test *p* = 0.039). Genes mapped to CpG sites with PC2 factor loading ≥ | 0.02| (*n* = 554 probes which mapped to 341 genes; Supplementary Table 1) include *TTC12* [loading = −0.098 (top of the list); together with *NCAM, ANNK1* and *DRD2*, forming a gene cluster which is associated with substance dependence (Mota et al., 2015)], *GABBR1* (loading = −0.087; a GABA_*B*_ receptor subunit), *CSNK1D* [loading = −0.068; related to circadian rhythm and lithium response in BD (Geoffroy et al., 2018)], and *KCNC4* (loading = 0.044; a voltage-gated potassium channel). These results were not driven by CpG sites located in CpG island, shelf, or shore, since after restricting to CpG sites only within these regions, neither PC1 nor PC2 was associated with rapid cycling (*p* > 0.05; Supplementary Figure 3).

### Cell-Type Composition in cfDNA Samples

Using the cell-type methylomic deconvolution algorithm and reference atlas by Moss et al. (2018), we estimated the distribution of cfDNA origins among 26 reference cell types/tissues (Table 2). An average of 71% of qualified probes matched the reference atlas probes of each tissue/cell type and the cfDNA tissue/cell type compositions were significantly different between our samples and Moss et al. (2018) in the left atrium, prostate, vascular endothelial cells, colon epithelial cells, lung cells, kidney, hepatocytes, bladder, and head and neck larynx (*p* < 0.001; Supplementary Table 2). Most of the blood cell types (monocytes, NK-cells, neutrophils, and erythrocyte progenitors) had the lowest probe overlapping rates (∼54%), while breast, pancreatic acinar cells, upper GI tract, vascular endothelial cells, hepatocytes, and cortical neurons had the highest overlapping rates (79–81%; Supplementary Table 2). Table 2 shows the comparison of estimated cfDNA tissue/cell type of origin distribution between rapid cycling groups. Overall, the most prevalent estimated cfDNA tissue/cell type of origins were blood cells (neutrophils, erythrocyte progenitors, monocytes, and lymphocytes), which accounted for 82.6% of total cfDNA origins. Estimated cfDNA origins from the left atrium (3.3%), cortical neurons (2.9%), vascular endothelial cells (2.4%), and colon epithelial cells (2.1%) were also substantially present. Between rapid cyclers and non-rapid cyclers, no significant differences in estimated cfDNA cell/tissue types of origin were found (*p* > Bonferroni-adjusted α = 0.05/26 = 0.001923; individual sample compositions are shown in Supplementary Figure 4), hence no adjustment in tissue/cell type origin distribution on the methylome analysis was conducted.

**TABLE 2:** Comparison of estimated cfDNA tissue/cell type origin distribution between BD rapid cyclers and non-rapid cyclers.

Cell types/Tissues	Total	Percentage (SD)		*P*
		
		Non-rapid cyclers (*n* = 47)	Rapid cyclers (*n* = 46)	
Neutrophils	35.4%	37.0% (18.4%)	33.9% (16.9%)	0.431
Erythrocyte progenitors	25.0%	24.0% (14.0%)	26.0% (13.4%)	0.557
Monocytes	12.6%	12.9% (5.3%)	12.4% (5.0%)	0.833
Lymphocytes	9.6%	9.3% (6.2%)	9.9% (5.9%)	0.645
NK cells	5.3%	5.0% (2.7%)	5.7% (3.2%)	0.375
Left atrium	3.3%	2.9% (2.3%)	3.7% (2.4%)	0.153
Cortical neurons	2.9%	2.8% (1.4%)	2.9% (1.6%)	0.926
Prostate	2.6%	2.6% (1.4%)	2.6% (1.5%)	0.812
Vascular endothelial cells	2.4%	2.4% (2.4%)	2.4% (2.2%)	0.981
Colon epithelial cells	2.1%	2.1% (0.9%)	2.1% (0.9%)	0.741
B cells	1.7%	1.5% (2.3%)	1.9% (2.7%)	0.386
CD8^+^ T cells	1.4%	1.4% (1.9%)	1.4% (2.1%)	0.990
Lung cells	1.3%	1.4% (1.2%)	1.2% (1.1%)	0.410
CD4^+^ T cells	1.2%	1.5% (3.6%)	0.9% (2.0%)	0.920
Kidney	0.9%	1.0% (1.0%)	0.9% (1.1%)	0.472
Hepatocytes	0.5%	0.4% (0.7%)	0.6% (0.9%)	0.041
Pancreatic acinar cells	0.3%	0.2% (0.3%)	0.5% (0.6%)	0.010
Adipocytes	0.2%	0.0% (0.3%)	0.4% (2.6%)	0.547
Pancreatic beta cells	0.2%	0.3% (0.5%)	0.2% (0.4%)	0.157
Thyroid	0.2%	0.2% (0.5%)	0.2% (0.5%)	0.686
Pancreatic duct cells	0.1%	0.1% (0.3%)	0.1% (0.3%)	0.831
Bladder	0.1%	0.2% (0.6%)	0.1% (0.3%)	0.681
Breast	0.1%	0.1% (0.5%)	0.1% (0.3%)	0.942
Uterus cervix	0.0%	0.0% (0.1%)	0.0% (0.0%)	1.000
Head and neck larynx	0.0%	0.0% (0.0%)	0.0% (0.0%)	–
Upper GI	0.0%	0.0% (0.0%)	0.0% (0.0%)	–

*Cell types and tissues are listed in descending order of their proportions in the cfDNA samples.*

### cfDNA Methylomic Differences Between Rapid Cyclers and Non-rapid Cyclers

**FIGURE 2:**
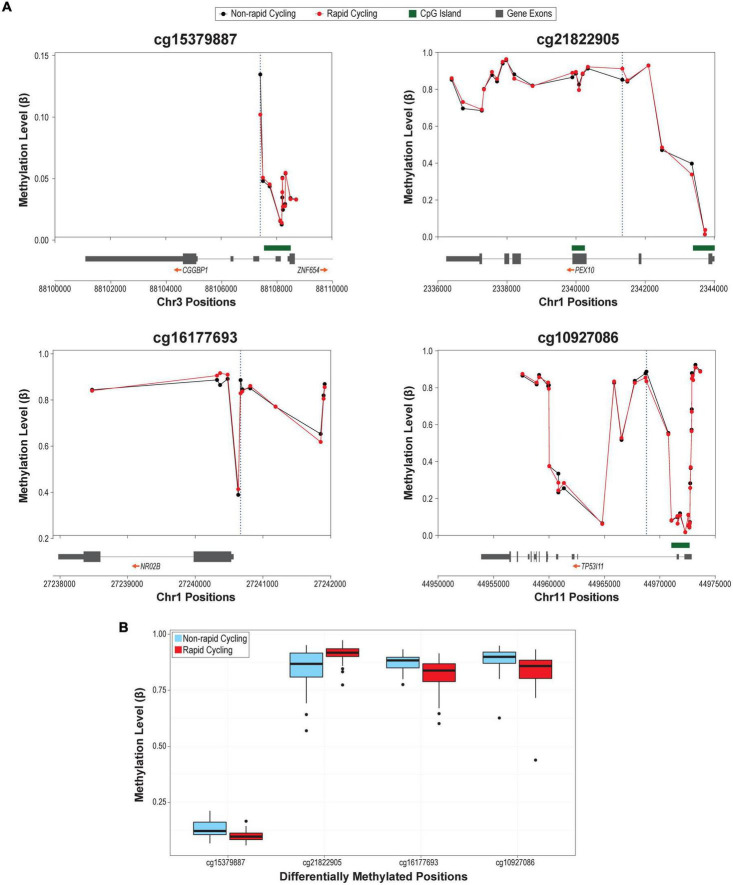
**(A)** Regional plots of the top differentially methylated probes (DMPs; *p* < 1E-05). Blue dotted vertical lines mark the genomic locations of the most significantly differentially methylated CpGs in that region. **(B)** Box plots of the methylation levels of the four DMPs differed between rapid cyclers and non-rapid cyclers at *p* < 1E-05.

The overall cfDNA methylomic differences between rapid cyclers and non-rapid cyclers were subtle as demonstrated in the heatmap (Figure 1B). A Manhattan plot of the genome-wide cfDNA methylomics comparison is shown in Figure 1C. No significant between-group DMPs (*q* > 0.15; top 2000 CpGs are listed in Supplementary Table 3) or DMRs (Stouffer *p* > 0.05; listed in Supplementary Table 4; top ten DMRs are shown in Supplementary Figure 5) were found. The top CpG sites with the most significant between-group differences at *p* < 1E-05 (cg15379887, cg21822905, cg16177693, and cg10927086) were located in *CGGBP1*, *PEX10*, *NR0B2*, and *TP53I11*. Rapid cyclers had relatively lower methylation levels in the promoter and/or 5′UTR of *CGGPB1* (north CpG shore), *NR0B2* (south CpG shore), and *TP53I11* (north CpG shelf), while they had relatively higher methylation levels in the body of *PEX10* (open sea; Figure 2).

We performed GSEA on genes with CpG sites that were different between groups at *p* < 0.05 (including 23,508 probes that mapped to 10,798 genes). We found significant enrichment in 26 GO gene sets, which are related to synapses, neurons, neurotransmissions, and nervous system development, and in three KEGG gene sets, which are involved in parathyroid regulation and calcium signaling (FDR < 0.05; Table 3). The list of genes and CpG sites mapped to these pathways is shown in Supplementary Table 5.

**TABLE 3:** Pathway enriched by genes with CpG sites that differed between rapid cycling groups at *p* < 0.05.

Pathway names	Gene hits	#CpG sites	*p*	FDR
**GO pathways**				
Neuron part	1,701	1,037	5.28E-08	< 0.001
Postsynapse	610	408	5.53E-08	< 0.001
Synapse	1,163	736	6.29E-08	< 0.001
Synapse part	931	596	1.44E-07	0.001
Nervous system development	2,333	1,391	1.54E-07	0.001
Neuron projection	1,291	794	3.13E-07	0.001
Cell projection part	1,443	874	2.21E-06	0.006
Plasma membrane bounded cell projection part	1,443	874	2.21E-06	0.006
Plasma membrane bounded cell projection	2,071	1,226	3.89E-06	0.010
Protein dimerization activity	1,298	740	1.10E-05	0.024
Neuron differentiation	1,353	830	1.32E-05	0.024
Protein homodimerization activity	848	501	1.44E-05	0.024
Enzyme binding	2,172	1,261	1.49E-05	0.024
Cytoskeletal protein binding	964	601	1.70E-05	0.024
Negative regulation of cellular process	4,719	2,564	1.88E-05	0.024
Somatodendritic compartment	828	515	1.91E-05	0.024
Cell junction	1,289	788	1.99E-05	0.024
Axon	605	390	2.04E-05	0.024
Cell projection	2,146	1,261	2.06E-05	0.024
Neuron to neuron synapse	347	238	2.14E-05	0.024
Neurogenesis	1,607	970	2.18E-05	0.024
Plasma membrane region	1,183	719	2.38E-05	0.025
Generation of neurons	1,508	914	3.04E-05	0.030
Cell leading edge	400	269	3.26E-05	0.031
Glutamate receptor binding	46	40	3.43E-05	0.031
Cell morphogenesis involved in differentiation	739	478	4.33E-05	0.038
Neuron development	1,100	683	4.61E-05	0.039
Axon guidance	275	194	5.13E-05	0.042
Cell-cell signaling	1,615	947	5.54E-05	0.044
Cytoskeleton organization	1,308	780	5.90E-05	0.045
Receptor complex	388	253	6.29E-05	0.046
Actin binding	425	280	6.42E-05	0.046
Dendrite	609	387	7.08E-05	0.047
Rab GTPase binding	166	116	7.13E-05	0.047
Synaptic membrane	429	282	7.28E-05	0.047
Neuron projection guidance	276	194	7.47E-05	0.047
**KEGG**				
Amoebiasis	97	73	8.00E-06	0.001
Parathyroid hormone synthesis, secretion and action	105	82	8.23E-06	0.001
Calcium signaling pathway	237	160	1.84E-04	0.021

### Leave-One-Out Cross-Validation of Top Between-Group cfDNA Methylomic Differences

We performed leave-one-out cross-validation analysis to verify that our top between-group DMPs were not driven by individuals with exceptionally high or low methylation levels. For the four DMPs with the top methylation level differences between rapid cyclers and non-rapid cyclers, we iteratively removed one individual and repeated the unpaired contrasts with adjustment of age and sex until all subjects had been excluded once. From each iteration, the log_2_ fold changes and *p*-values of the target DMPs were recorded. The log_2_ fold changes and *p*-values reported on the top four DMPs in our initial analysis were extremely close to the mean values obtained from the leave-one-out contrasts (Supplementary Table 6), thus confirming our top findings were not driven by extreme methylation values in certain samples.

## Discussion

In this study, we used a microarray method to detect plasma cfDNA methylation levels to examine the cfDNA methylomic differences that were associated with rapid cycling in BD. We found global differences in methylation profiles between BD rapid cyclers and non-rapid cyclers, four CpG sites with differential methylation levels between BD rapid cyclers and non-rapid cyclers at *p* < 1E-05 (not genome-wide significant), and significant enrichment in pathways related to neurons and synaptic functions among the top CpG sites that differed between the groups. To our knowledge, this is the first investigation of cfDNA methylomics as potential biomarkers for BD rapid cycling, thereby marking an example of cfDNA application in psychiatric diagnosis and providing insights into amelioration of rapid mood cycling in BD.

Most cfDNA methylomic studies used sequencing-based methods such as whole-genome bisulfite sequencing, reduced representation bisulfite sequencing, and methylated CpG tandem amplification and sequencing (Zeng et al., 2018), while a few studies used microarray analysis (Gallardo-Gómez et al., 2018; Gordevicius et al., 2018). Compared to sequencing-based methods, microarrays have much lower coverage but provide an economic genome-wide platform that screens CpG sites located at gene bodies and regulatory elements (Huang and Wang, 2019) and the required amount and quality of input DNA are generally less demanding. In our study, the low locus detection rates (<99%) and high exclusion rate of CpG probes from analysis (∼45%) might be caused by insufficient input DNA, exacerbated by the biased DNA fragmentation pattern in cfDNA which can reduce coverage in transcription start sites and exon boundaries (Ma et al., 2017). In future mood disorder studies where other origins of organ damage and the presence of tumors can be excluded, strategies for increasing input cfDNA amount would be needed to increase cfDNA to boost experimental performance, such as pooling of cfDNA samples from multiple well-matched samples (Gallardo-Gómez et al., 2018).

The estimated proportions of tissues and cell types that contributed to the cfDNA in our samples were slightly different from those reported by Moss et al. (2018), which may be due to differences in participants’ demographic and clinical characteristics, the cfDNA preparation procedures employed, and the quantity of input cfDNA applied onto the EPIC arrays. Participants in Moss et al. (2018) were healthy individuals aged 19–30 years and ≥ 75 years, while our BD participants were mainly middle-aged (20–79 years; mean 45 years). Moreover, they used pooled cfDNA samples generated by mixing cfDNA samples of several subjects (7–19 samples/pool) classified by age and sex until the sample reaching 250 ng cfDNA which was the recommended input DNA amount of the Illumina methylomic arrays (Moss et al., 2018). Moss et al. (2018) evaluated the performance of the deconvolution algorithm using 100 and 50 ng of cfDNA, i.e., amounts much less than manufacturer-recommended 250 ng, and found their predicted tissue/cell type distributions correlated with the distributions obtained with the recommended amount at *r* > 0.99 and > 0.90, respectively. Therefore, we projected that the reproducibility of applying the Moss et al. (2018) method to our microarray data would be lower but still satisfactory. Relative to the cfDNA tissue/cell type of origin proportions in healthy individuals reported by Moss et al. (2018), our data obtained in BD patients were largely similar for most tissues/cell types but dissimilar in some notably, for example, we found a significantly higher proportion of vascular endothelial cells (Supplementary Table 2). These differences may be attributed to BD medications, medical comorbidities of BD such as the increased risk of cardiovascular diseases (Goldstein et al., 2020), and/or the aforementioned technical differences between studies that affected the performance of the tissue/cell type estimation.

The top differentially methylated sites between rapid cyclers and non-rapid cyclers were *CGGBP1*, which encodes for CGG triplet repeat-binding protein 1, a nuclear protein that selectively binds to unmethylated CGG trinucleotide repeats. The hypomethylation in a CpG site which is located in the *CGGBP1* 5′UTR and at the north shore of a CpG island implies an upregulation of gene expression (Martino and Saffery, 2015). CGGBP1 is expressed ubiquitously across human tissues. It was found to regulate gene transcription and participate in cell growth and proliferation with cytoprotective properties (Singh and Westermark, 2015). Its specific roles in transcription regulation include, but are not limited to, shielding unmethylated CGG repeats from methylation (Deissler et al., 1996), histone modification (Stelzl et al., 2005; Patel et al., 2019), and acting as a *trans-*regulator of RNA polymerase II-transcribed genes (Agarwal et al., 2016). CGGBP1 deficiency results in cell cycle arrest at the S phase and G2/M phase in normal cells (Singh and Westermark, 2011). Notably, *CGGBP1* as a D-box-containing protein (D-box is a *cis*-regulatory element known to associate with zenith expression of genes during the “active phase”) has been found to demonstrate circadian variations in its expression level in intestinal epithelial cells in response to an unusual restricted feeding schedule in mice, thus demonstrating that *CGGBP1* as a component of the peripheral circadian clock (Mukherji et al., 2015). Reduced *CGGBP1* gene expression had been reported in the peripheral blood of patients with post-traumatic stress disorder (Uddin et al., 2011). Therefore, altered *CGGBP1* may be a critical indicator of the misalignment between the central and peripheral circadian clocks which could progressively induce metabolic syndrome (Mukherji et al., 2015) and increase the risk of mood disorders (Baron and Reid, 2014). However, due to the scarcity of studies on the association between *CGGBP1* and psychiatric disorders, the involvement of *CGGBP1* in mood disorders is yet to be identified.

*PEX10* encodes peroxisomal biogenesis factor 10, a ubiquitously expressed peroxisomal matrix protein. Peroxisomes are organelles that are critical for fatty acid and phospholipid metabolisms, such as very-long-chain fatty acid catabolism and the biosynthesis of docosahexaenoic acid (DHA) and plasmalogens which are essential for normal brain development and functioning. Peroxisome dysfunction has been associated with neurodegenerative disorders (Jo and Cho, 2019) and mood disorders (McNamara et al., 2010). Lower erythrocyte DHA level was observed in BD1 patients and found to be associated with the onset of the first mania episode (McNamara et al., 2015) and increased risk of BD1 development (McNamara et al., 2016a), but contrasting results have also been reported (McNamara et al., 2016b). Therefore, the association between peroxisomal dysfunction and BD remains uncertain. *PEX10* mutations can cause Zellweger syndrome (Okumoto et al., 1998). Although *PEX10* has not been extensively studied in the brain, *Pex10* deficiency in mice resulted in reduced Schwann cell count and poor integrity of axon and synapse in the spinal cord (Hanson et al., 2014). Here, we found a CpG site in *PEX10* gene body was hypermethylated in the rapid cycling group, implying a potentially altered *PEX10* gene expression level may be associated with BD rapid cycling. For example, the altered PEX10 levels may affect fatty and phospholipid acid metabolisms, which are important to brain health, thereby precipitating mood instability. However, it is unclear which tissue was responsible for this difference and whether the observed changes were functionally significant.

*NR0B2* encodes nuclear receptor subfamily 0 group B member 2 (a.k.a. short heterodimer partner), an orphan nuclear receptor that interacts with receptors of estrogen, retinol, bile acid, and thyroid hormone to suppress the transcriptional activity of these nuclear receptors (Seol et al., 1997; Ma and Patti, 2014) as well as with peroxisome proliferator-activated receptor (PPAR) α and γ (Kassam et al., 2001; Nishizawa et al., 2002). *NR0B2* is mostly expressed in the gastrointestinal tract (stomach, duodenum, small intestine, and colon) and liver, and to a lesser extent in the gall bladder, spleen, pancreas, and heart. NR0B2 is involved in the regulation of bile acid synthesis (Yang et al., 2014), bile salt secretion (Neimark et al., 2004), inflammation (Maguire et al., 2017; Zhou et al., 2018), insulin secretion and pancreatic β cell survival (Suh et al., 2004; Noh et al., 2013), as well as lipid metabolism in liver and brown adipocytes (Wang et al., 2005; Khristi et al., 2019). Our finding of a lower methylation level in the promoter region of *NR0B2* suggests that an increase in *NR0B2* expression in some tissues may be related to BD rapid cycling. Since *NR0B2* is involved in lipid and glucose metabolisms and inflammation, dysfunctionality in these processes may be associated with the risk of rapid mood cycling, this coincides with the higher rates of rapid cycling observed in BD patients with diabetes mellitus or insulin resistance (Ruzickova et al., 2003; Calkin et al., 2015).

*TP53I11* encodes tumor protein P53 inducible protein 11 and are expressed ubiquitously in human tissues. It has been studied almost exclusively in oncology and information on its association with mood disorders is absent. TP53I11 was found to promote apoptosis by binding to DNA (Liang et al., 2004; Xiong et al., 2007) as well as inhibit tumor metastasis (Xiao et al., 2019). Its association with BD rapid cycling remains to be uncovered.

In the list of genes with top differentially methylated CpG sites (*p* < 0.05), nearly all significantly enriched GO gene sets were related to the structure, generation, and development of neurons as well as the structure and functioning of synapses, suggesting that plasma cfDNA may contain brain-derived DNA and those methylation levels could reflect the presence of rapid cycling feature in BD. However, this postulation needs empirical support from tissue-specific methylome analyses and also proof that neuronal and synaptic dysfunctions contribute to rapid cycling in BD. Among various neurotransmitter systems, the “glutamate receptor binding” pathway was significantly enriched, thereby reiterating the importance of the glutamatergic system in regulating rapid mood cycling in BD found in previous studies (Michael et al., 2009; Veldic et al., 2019). Another interesting enriched gene set was “Rab GTPase binding” as Rab GTPases are critical players in vesicular trafficking between the cell body and axonal presynaptic and dendritic postsynaptic terminals and in synaptic vesicle docking and exocytosis at the presynapse, regulating neuronal development and polarization, presynaptic neurotransmitter release, and postsynaptic membrane composition (Mignogna and D’Adamo, 2018). Two significantly enriched KEGG gene sets, “parathyroid hormone, synthesis, secretion and action” and “calcium signaling pathway,” pointed toward calcium homeostasis and the effects of calcium on various cell types, such as muscle contraction and long-term potentiation and depression related to learning and memory. Although the relationship between parathyroid hormone and mood is unclear, parathyroid hormone can cross the blood-brain barrier and parathyroid receptors are found throughout the human brain (Lourida et al., 2015). A recent study reported that serum parathyroid levels of BD patients were correlated positively with the total number of mood episodes and suicide attempts but negatively with the age of onset (Steardo et al., 2020). Notably and paradoxically, the use of lithium is known to induce hyperparathyroidism and hypercalcemia (Albert et al., 2013).

A few limitations need to be noted. Firstly, the blood samples from which the plasma cfDNA was derived were not collected in specific mood states (mostly during euthymia and depressed states for ethical reasons) or the change between mood states. However, obtaining biospecimens during or around the time of mood state is limited by practical and ethical reasons. Secondly, the organs and cell types responsible for the cfDNA detected in the samples could not be ascertained. We have adopted the method by Moss et al. (2018) for predicting the distribution of tissues/cell types of origin in cfDNA samples and found a substantial percentage of cfDNA with cortical neuronal origin in our samples and no significant differences in cfDNA tissues/cell types of origin distribution between groups. However, to determine that a tissue/cell type contributes to a certain cfDNA methylomic difference, methods that can detect the methylation pattern in each cfDNA fragment (e.g., sequencing) coupled with an advanced deconvolution algorithm would be required. Validation in specific tissues and cell types, such as peripheral blood mononuclear cells, are needed to confirm the origins of our findings. Thirdly, since the sample size was small, the statistical power was limited and led to the lack of significant differentially methylated sites after multiple testing correction. Lastly, in the absence of a replication experiment, in order to verify that our top findings were not due to extreme methylation values in our samples, we conducted leave-one-out cross-validation and found that our initial reported statistics were not driven by a single outlier sample. Nevertheless, replication using an independent cohort of subjects would be needed to confirm our findings.

To conclude, this study is the first investigation of circulating cfDNA methylomic differences of BD patients with rapid cycling to provide support for the diagnostic application of circulating cfDNA methylomics. We demonstrated the use of a microarray method for plasma cfDNA methylome analysis and identified potential differences associated with BD rapid cycling. The differentially methylated CpGs were mostly within or near genes involved in pathways related to the nervous system and parathyroid. Our findings need to be replicated with a larger, independent cohort and followed up with a more definitive assignment of the cfDNA methylomic signals to tissues-of-origin.

## Data Availability Statement

The datasets presented in this article are not readily available because of research ethics protocol restriction. Requests to access the datasets should be directed to the corresponding author.

## Ethics Statement

The studies involving human participants were reviewed and approved by the Mayo Clinic Institutional Review Board. The patients/participants provided their written informed consent to participate in this study.

## Author Contributions

AM-CH and MV conceived the study. SW and BM provided advice on the study design and conducted the statistical analyses. AM-CH wrote the manuscript. MK, KR, ZS, TO, LW, and MF provided expert opinions on the study and critically reviewed the manuscript. All authors contributed to the article and approved the submitted version.

## Conflict of Interest

MF was a consultant (for Mayo Clinic) to Janssen, Mitsubishi Tanabe Pharma Corporation, Myriad, Sunovion, and Teva Pharmaceuticals; none of this funding contributed to any work carried out in this study. The remaining authors declare that the research was conducted in the absence of any commercial or financial relationships that could be construed as a potential conflict of interest.

## Publisher’s Note

All claims expressed in this article are solely those of the authors and do not necessarily represent those of their affiliated organizations, or those of the publisher, the editors and the reviewers. Any product that may be evaluated in this article, or claim that may be made by its manufacturer, is not guaranteed or endorsed by the publisher.
