# Supplementary material for: Plasma Cell-Free DNA Methylomics of Bipolar Disorder With and Without Rapid Cycling
Source: Front Neurosci. 2021 Nov 30;15:774037. doi: 10.3389/fnins.2021.774037 (PMC8669968; doi:10.3389/fnins.2021.774037)

Supplementary Materials

Supplementary Methods: Reasons of dropping probes 2
Supplementary Figure 1. PCA plots by substances of abuse 3
Supplementary Figure 2. PCA plots by psychotropic medications 4
Supplementary Figure 3. PCA plots by CpG islands, shores, and shelfs 5
Supplementary Figure 4. Cell type/tissue compositions of cfDNA 6
Supplementary Figure 5. Top ten differentially methylated region plots 7

Supplementary Methods: Reasons of dropping probes

**Reasons**	**No. of probes dropped**	**% Probes dropped**
Failing in one or more samples	293726	33.9%
Having SNP at CpG site	17675	2.0%
EPIC cross reactive site 1	34946	4.0%
EPIC cross reactive site 2	0	0.0%
EPIC cross reactive site 3	0	0.0%
EPIC overlapping CpG	471	0.05%
EPIC overlapping variants	21	0.002%
EPIC overlapping variants 2	55970	6.5%
**Total**	**402809**	**46.5%**

Supplementary Figure 1. PCA plot with sample highlighted according to substance abuse (green for not abusing/dependent on the substance, brown for abusing/dependent on the substance). X and Y chromosomes are excluded.

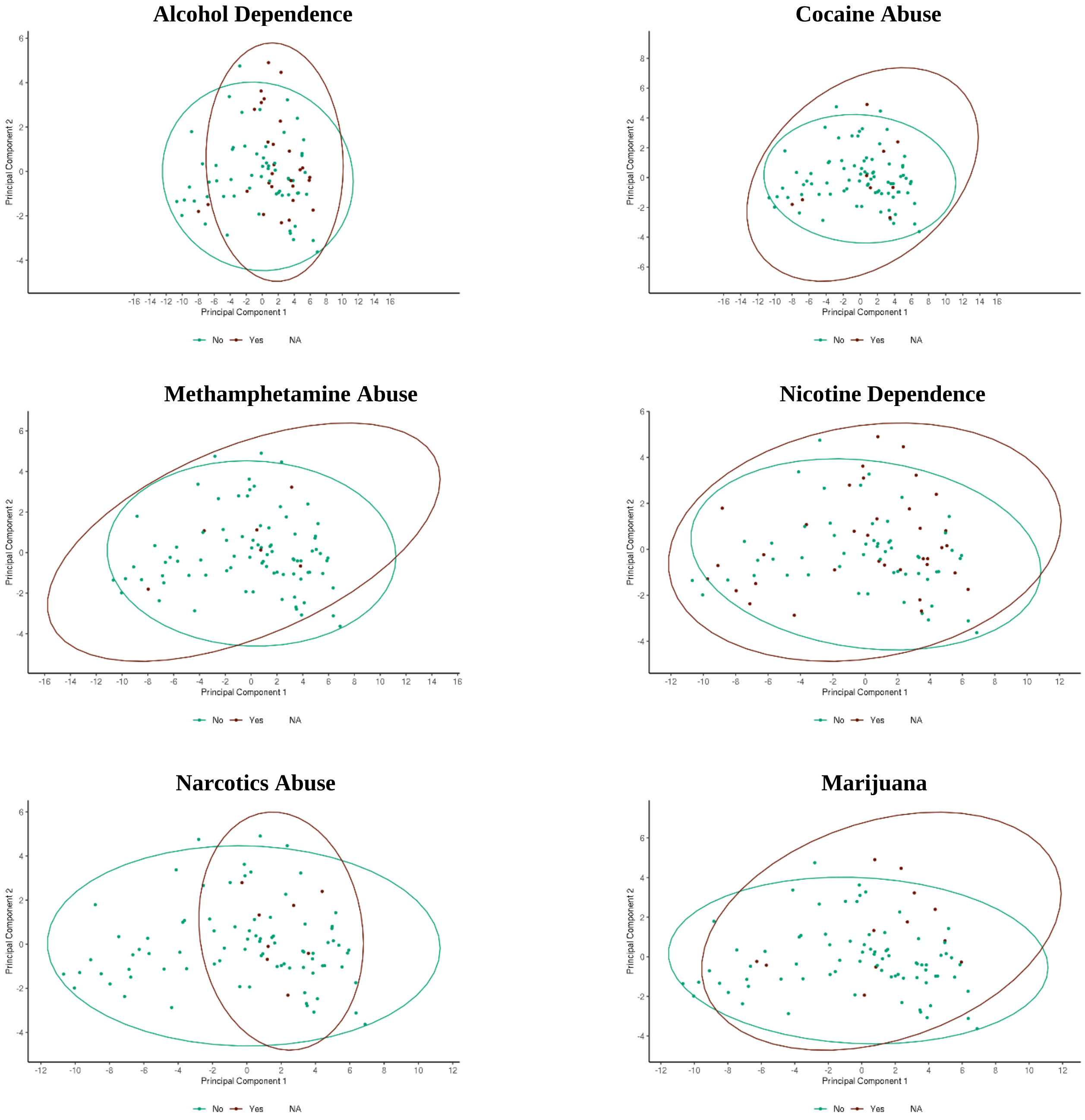

Supplementary Figure 2. PCA plot with sample highlighted according to psychotropic medication class (green for not taking the medication, brown for taking the medication). X and Y chromosomes are excluded.

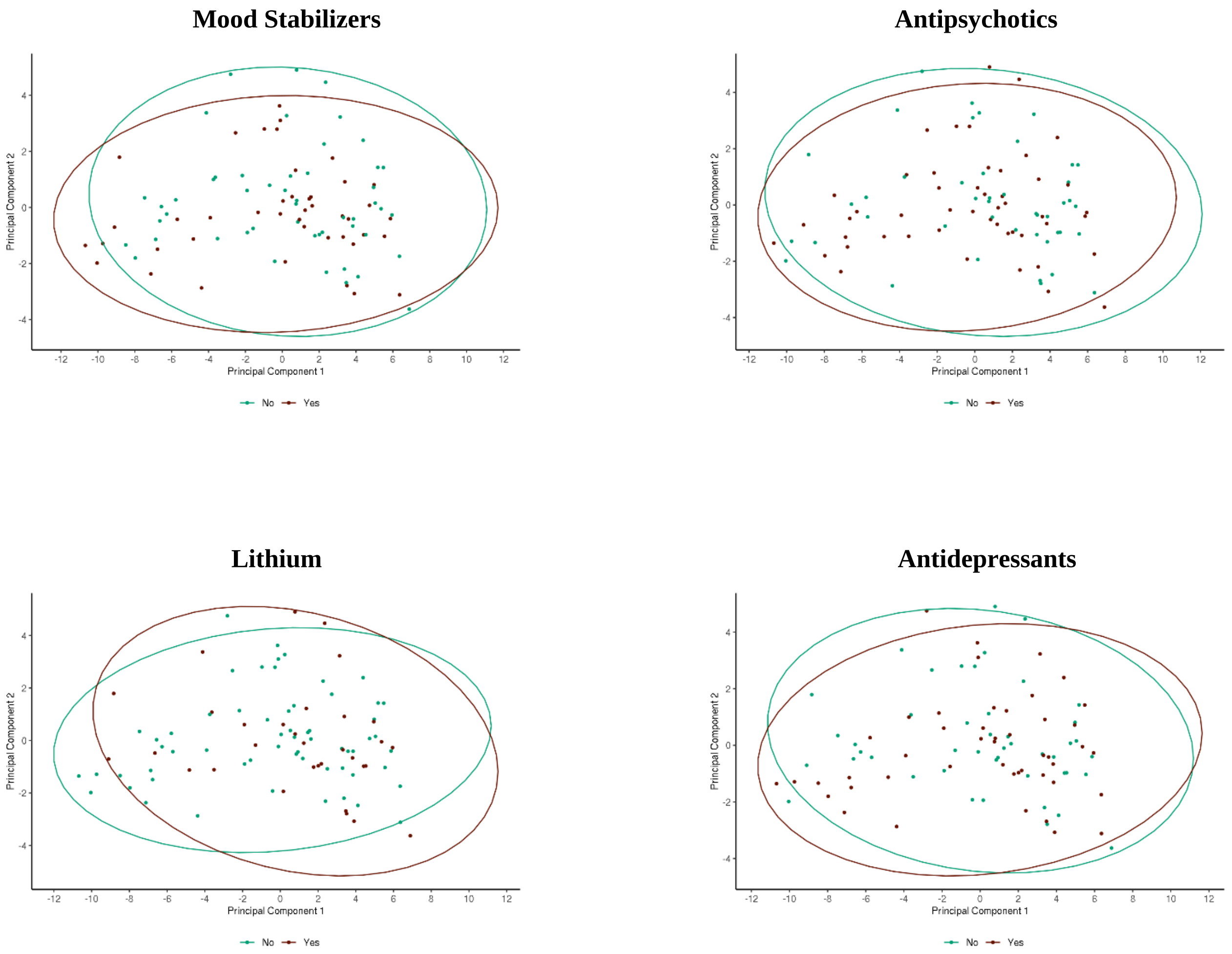

Supplementary Figure 3. PCA plot with sample highlighted according to rapid cycling subsetting according to location of the CpG sites (green for BD non-rapid cyclers, brown for BD rapid cyclers). X and Y chromosomes are excluded.

CpG Islands

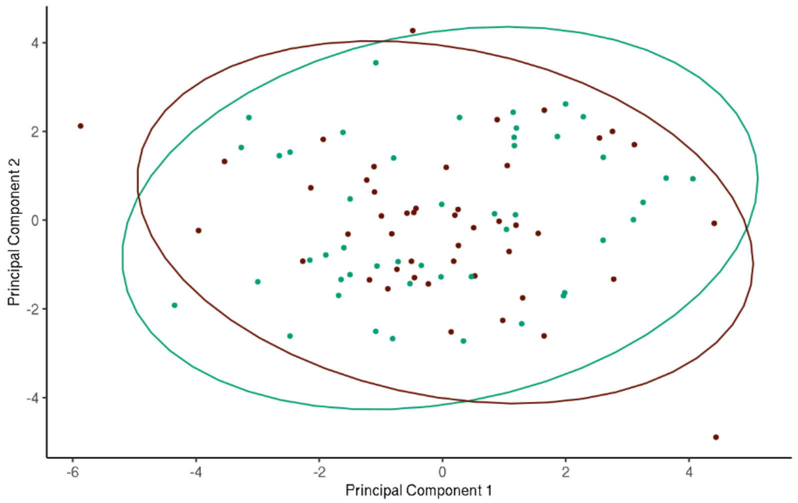

CpG Island Shelves

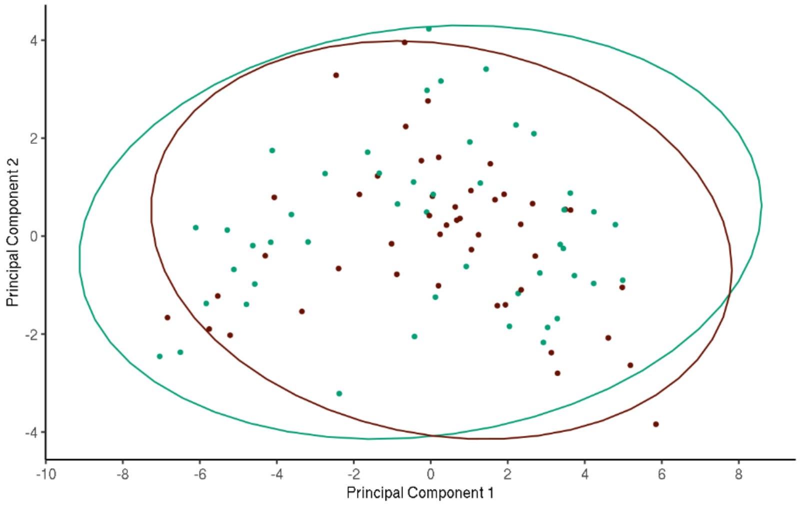

CpG Island Shores

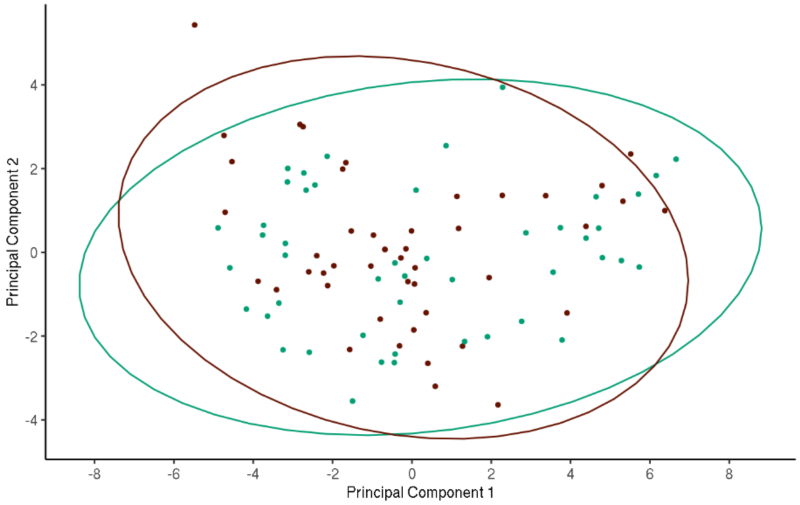

Supplementary Figure 4. Cell type/tissue compositions of cfDNA samples by rapid cycling groups.

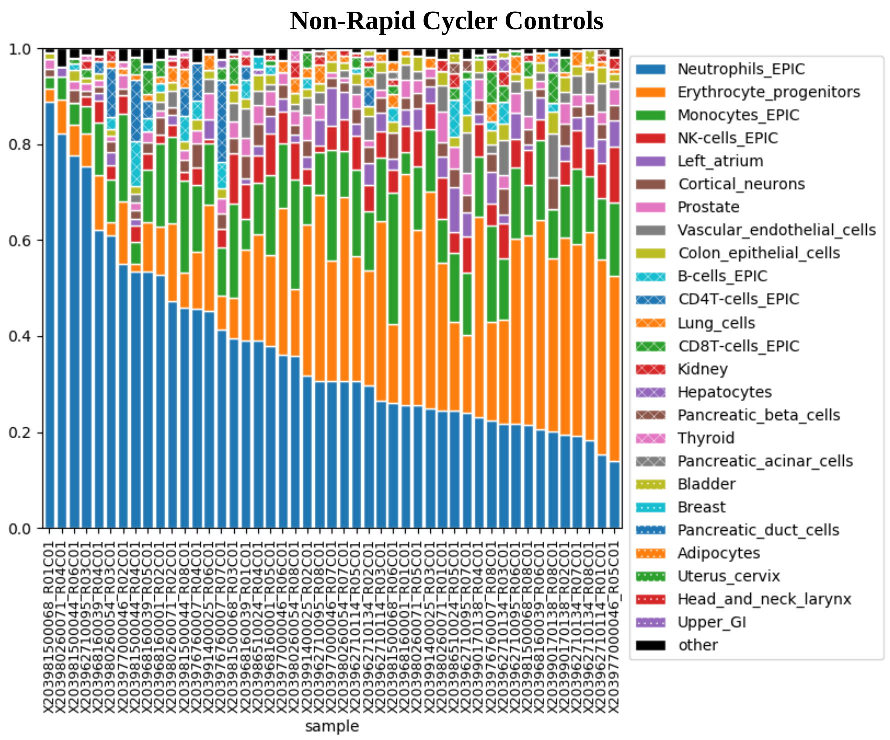

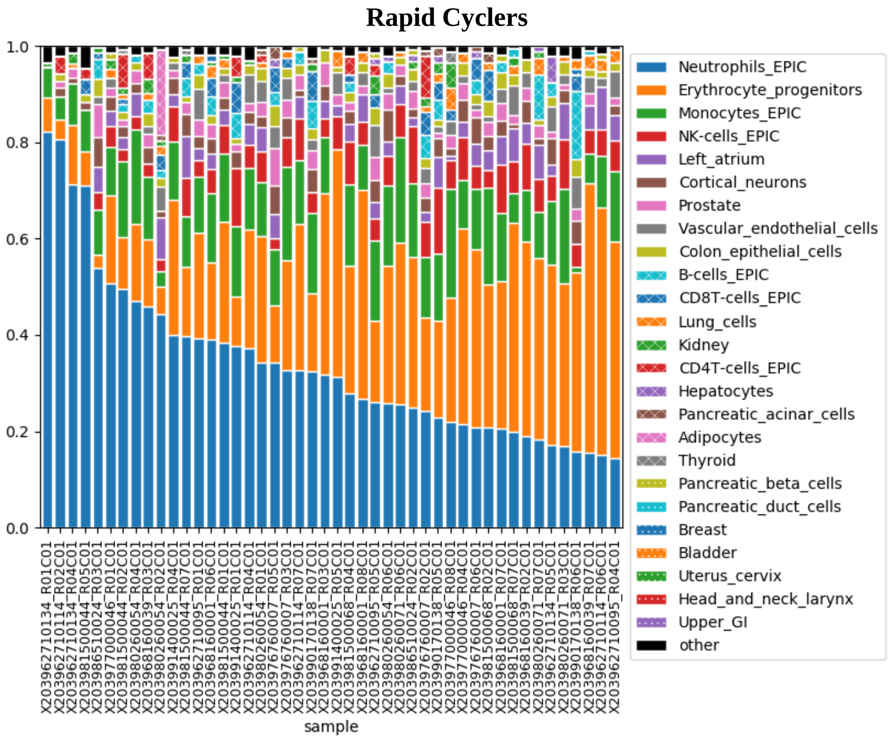

Supplementary Figure 5. Top ten differentially methylated region (DMR) plots.

TP53I11

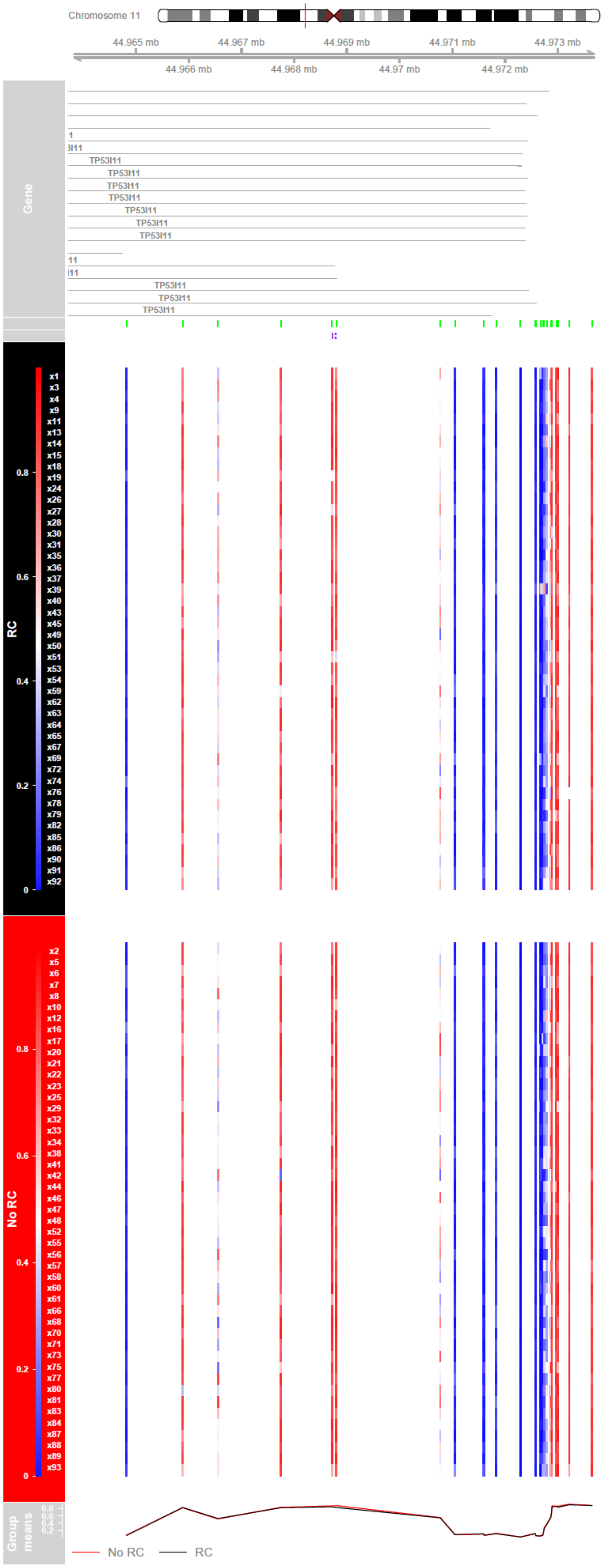

PACSIN1

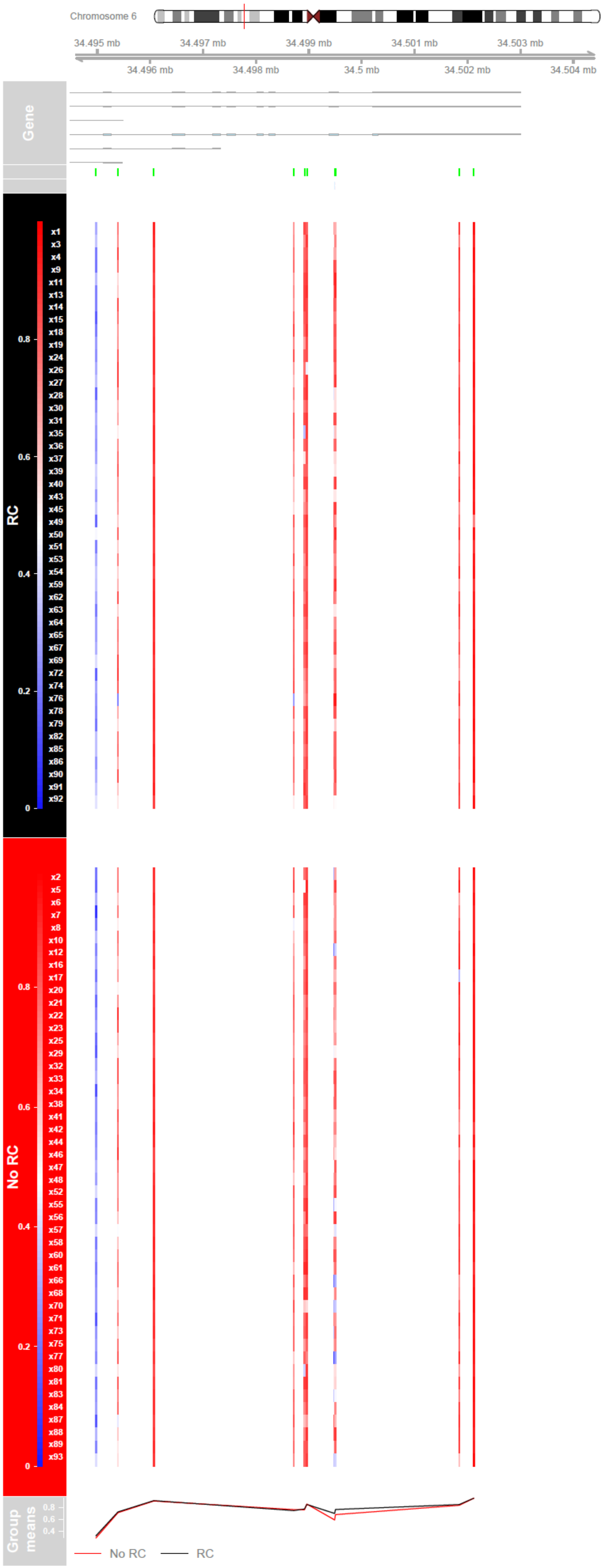

Supplementary Figure 5. (Cont'd) Top ten DMR plots.

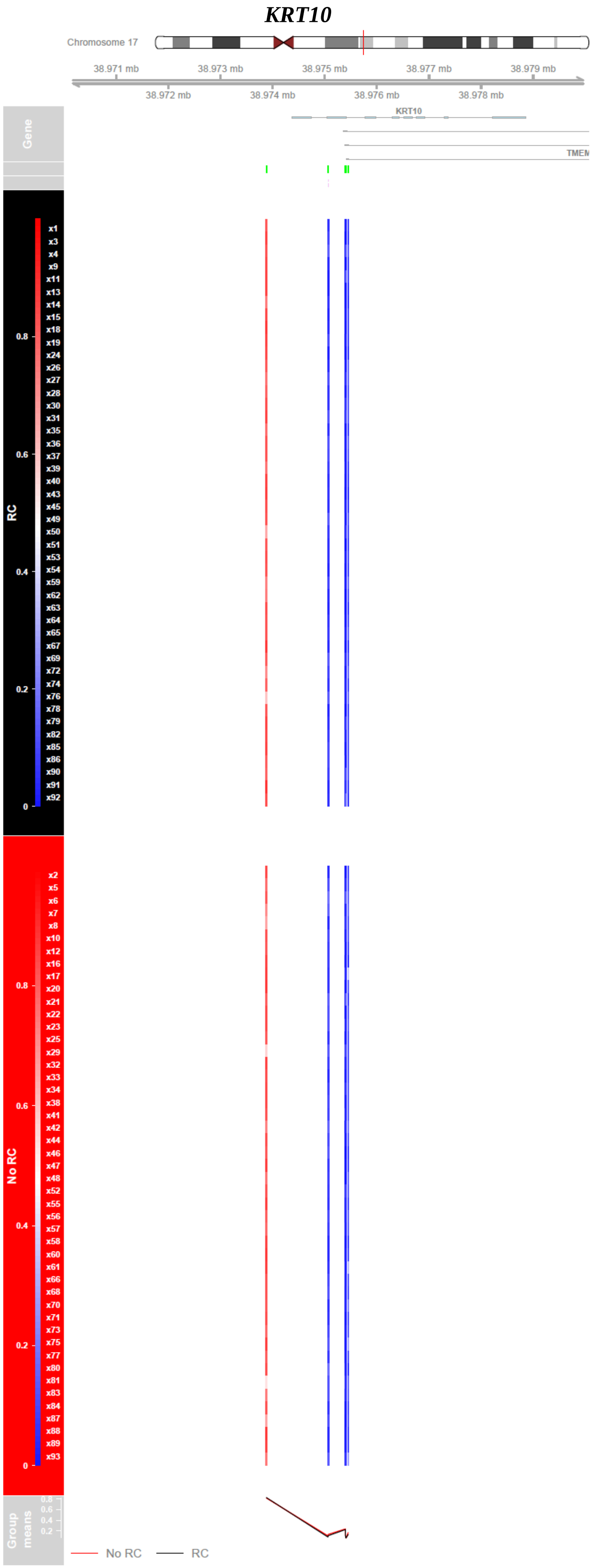

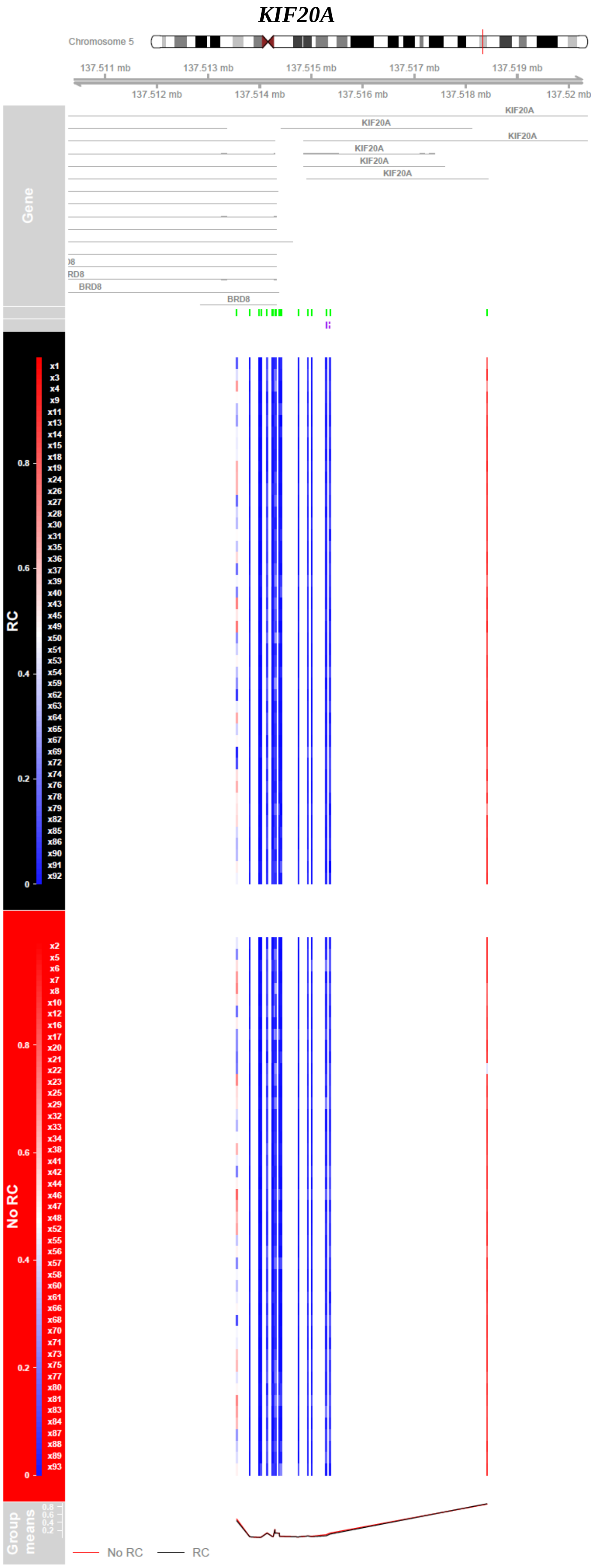

Supplementary Figure 5. (Cont'd) Top ten DMR plots.

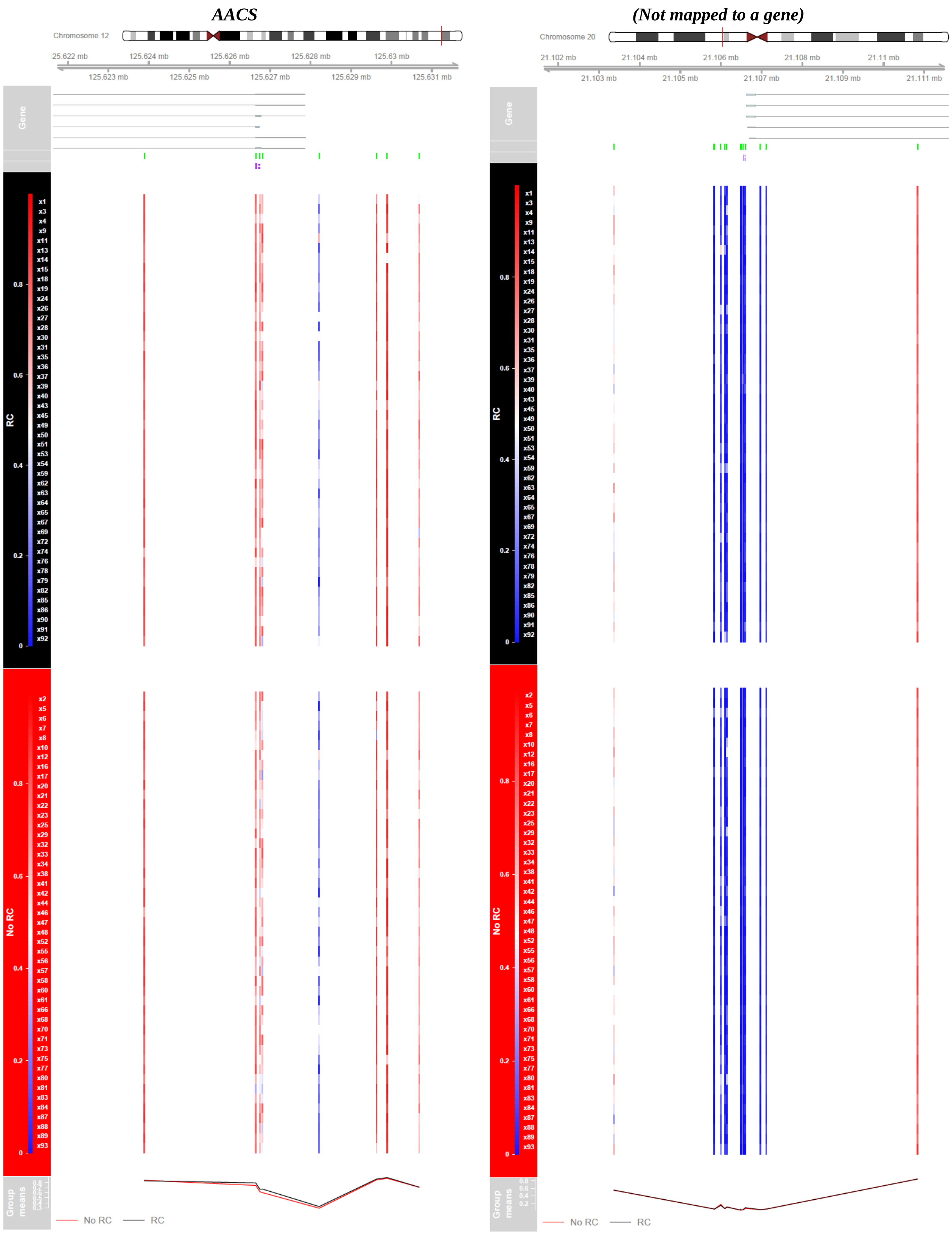

Supplementary Figure 5. (Cont'd) Top ten DMR plots.

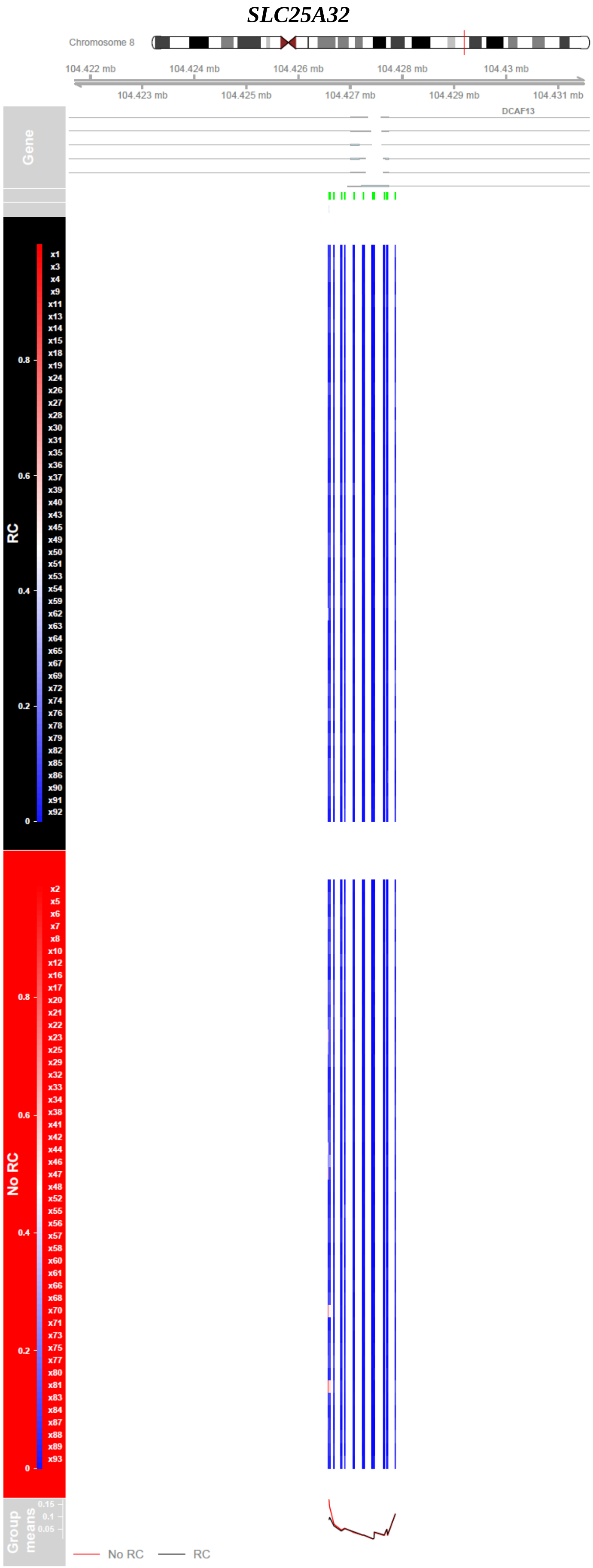

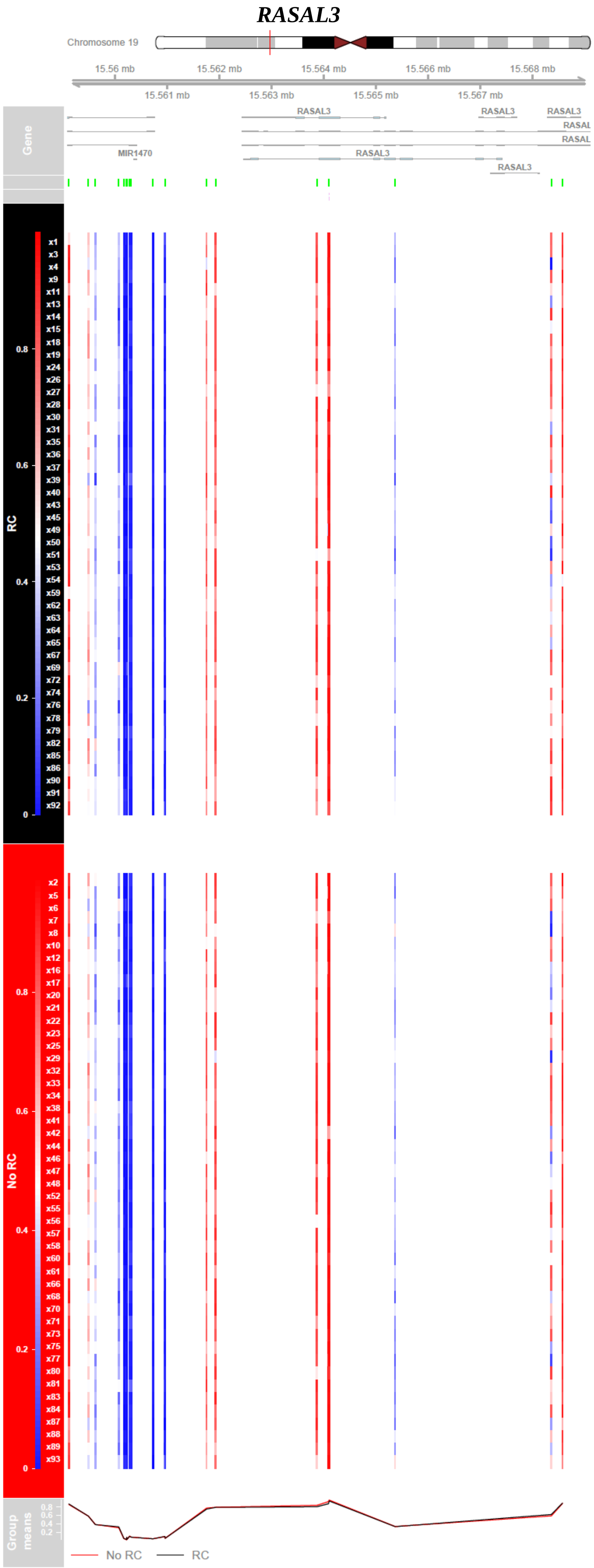

Supplementary Figure 5. (Cont'd) Top ten DMR plots.

RP11-103J8.1

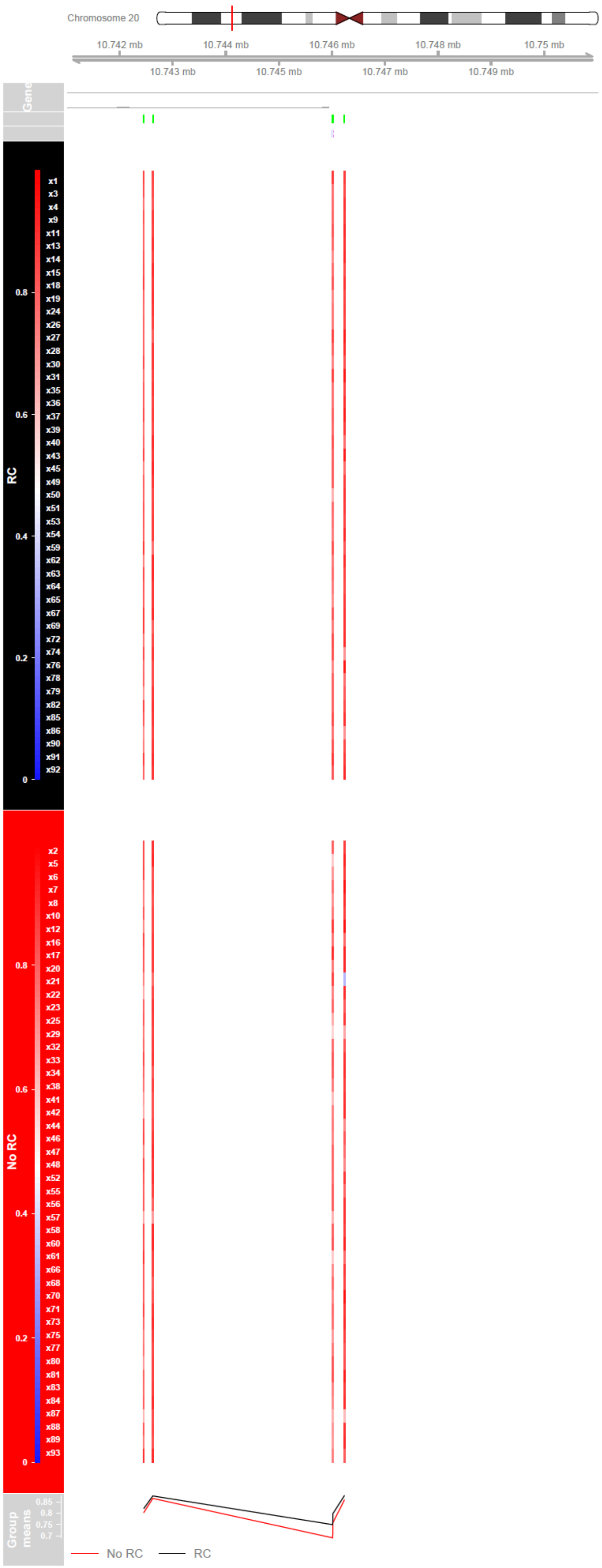

ACTN3, CTD-3074O7.2

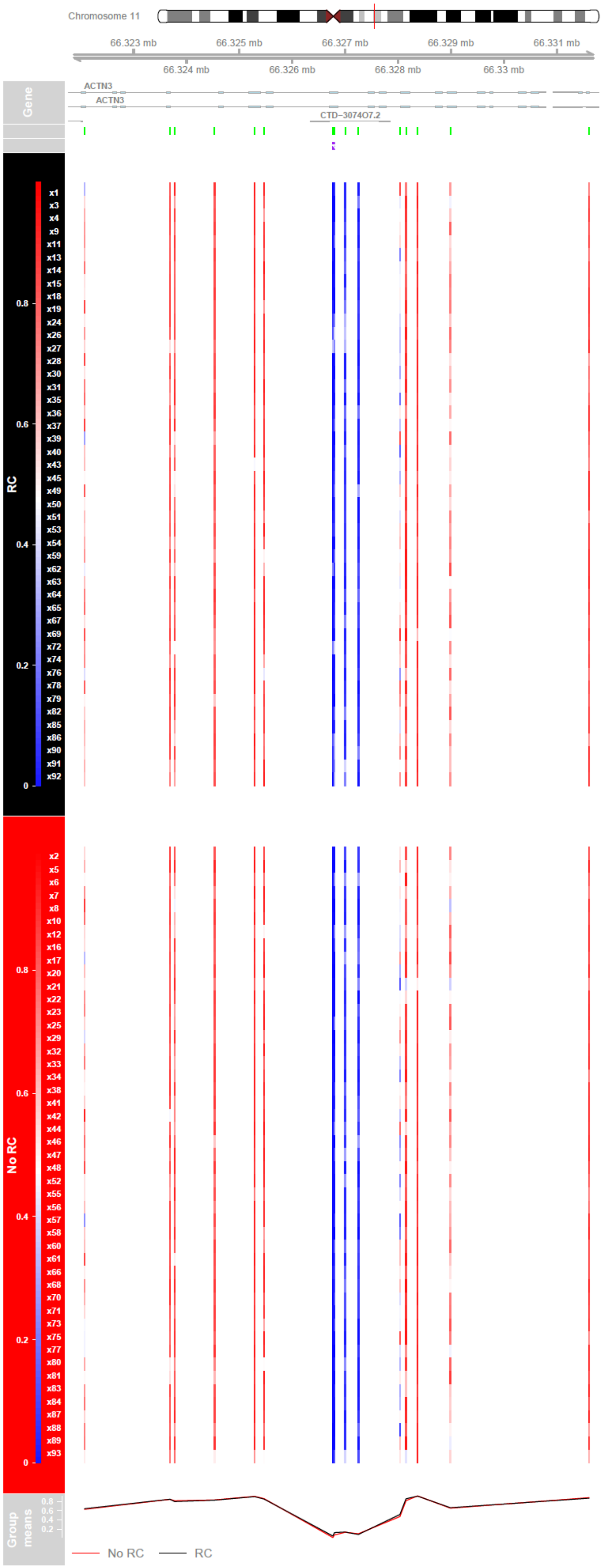

Supplement: Supplementary file 1 [file Data_Sheet_1.PDF]
